# Early Blood Biomarkers to Improve Sepsis/Bacteremia Diagnostics in Pediatric Emergency Settings

**DOI:** 10.3390/medicina55040099

**Published:** 2019-04-10

**Authors:** Emilija Tamelytė, Gineta Vaičekauskienė, Algirdas Dagys, Tomas Lapinskas, Lina Jankauskaitė

**Affiliations:** 1 Lithuanian University of Health Sciences, Medical Academy, 44307 Kaunas, Lithuania; emilijatam@gmail.com (E.T.); gineta.va@gmail.com (G.V.); algirdas.dagys@kaunoklinikos.lt (A.D.); tomas.lapinskas@lsmuni.lt (T.L.); 2 Department of Pediatrics, Hospital of Lithuanian University of Health Sciences Kauno Klinikos, 50161 Kaunas, Lithuania; 3 Department of Cardiology, Hospital of Lithuanian University of Health Sciences Kauno Klinikos, 50161 Kaunas, Lithuania

**Keywords:** pediatric, sepsis, biomarkers, NLR, PLT/MPV, diagnostics, emergency

## Abstract

*Background*: Sepsis is the leading cause of death in children worldwide. Early recognition and treatment are essential for preventing progression to lethal outcomes. CRP and Complete Blood Count (CBC) are the initial preferred tests to distinguish between bacterial and viral infections. Specific early diagnostic markers are still missing. *Aim*: To investigate diagnostic value of Neutrophil–Lymphocyte Ratio (NLR), Mean Platelet Volume (MPV) and Platelet–MPV ratio (PLT/MPV) to distinguish sepsis/bacteremia and viral infection. *Methods*: We conducted a retrospective data analysis of case records of 115 children from 1 month to 5 years of age. All cases were divided into two groups—sepsis/bacteremia (*n* = 68) and viral (*n* = 47) patients, and further subdivided according to the time of arrival into early or late (≤12 or 12–48 h post the onset of fever, respectively). Analysis of CBC and CRP results was performed. NLR and PLT/MPV were calculated. *Results*: Sepsis/bacteremia group demonstrated higher absolute platelets count (370.15 ± 134.65 × 10^9^/L versus 288.91 ± 107.14 × 10^9^/L; *p* = 0.001), NLR (2.69 ± 2.03 versus 1.83 ± 1.70; *p* = 0.006), and PLT/MPV (41.42 ± 15.86 versus 33.45 ± 17.97; *p* = 0.001). PLT/MPV was increased in early arrival sepsis/bacteremia infants (42.70 ± 8.57 versus 31.01 ± 8.21; *p* = 0.008). NLR and MPV were significantly lower in infants (≤12 months) with viral infection on late arrival (1.16 ± 1.06 versus 1.90 ± 1.25, *p* = 0.025 for NLR and 8.94 ± 0.95fl versus 9.44 ± 0.85fl, *p* = 0.046 for MPV). *Conclusion*: Together with standard blood biomarkers, such as CRP, neutrophils, or platelets count, PLT/MPV is a promising biomarker for clinical practice to help discriminate between viral disease or sepsis/bacteremia in all children, especially in early onset of symptoms. NLR and MPV could support exclusion of sepsis/bacteremia in late arrival cases.

## 1. Introduction

Pediatric sepsis remains one of the most common conditions causing death in infants and children worldwide [1,2,3,4,5]. To date, a child is diagnosed with sepsis when he or she presents with symptoms of systemic inflammatory response syndrome (SIRS) associated with a documented or suspected bacterial infection. However, based on clinical data alone, it is difficult to distinguish between a septic patient and a child with SIRS due to other causes. Fever, one of the most important SIRS components, is a very common reason in children to visit Emergency Department (ED) [6]. Majority of the febrile children will present with a self-limiting viral disease; however, some of them will have a severe bacterial infection (SBI) as sepsis [7,8]. The management of febrile infants have been an issue for nearly 30 years now, since most of the children with fever would only have a minor viral infection, while around 12% of infants aged <30 days or 9% of infants aged 30 to 90 days will have SBI [9]. Due to the immature innate and adaptive immune system, infants less than 3 months of age are at higher risk to develop SBI [10]. The risk increases with a younger age, since innate immune response is developing during the early stages of life and since innate immune response is the first line of defense mechanism against multiple microorganisms, thus making the infant most susceptible to SBI [11]. The prevalence of SBI declines with age [12]; still, the duration of fever and its height is greatly associated with sever bacterial infection [13]. The treatment of children who are ill-appearing and have obvious site of infection is clear. However, fever without a source (FWS) is of a big concern in ED settings. Moreover, children suffering from viral diseases or systemic autoimmune diseases can present with a SIRS. Additionally, there is often a 24–48 h period before a reason of SBI including sepsis can be fully confirmed or ruled out with laboratory testing. Thus, initial diagnosis of SIRS can lead to a further treatment “precaution” measurement when antibiotics are the primary choice of therapy [14]. On the other hand, delayed diagnosis and antibiotic treatment in case of sepsis or bacteremia can lead to severe complications as multiorgan failure or death [15,16]. Weiss et al. investigated the epidemiology of severe sepsis death, and found that it occurs between 3 and 7 days after the sepsis recognition, while later deaths were associated with multiple organ dysfunction syndrome (MODS), respiratory failure, or neurological conditions [17]. To date, the diagnostic pathways and different algorithms can fail to determine and accurately differentiate fever cause, as non-infectious diseases can present as SIRS as well. Moreover, overdiagnosis could lead to additional stressful testing procedures as blood culture or lumbar puncture. Besides, FWS or SIRS are often treated empirically without determining the etiological factor. Thus, it leads to an antibiotic overuse contributing to global antimicrobial resistance issue [18].

Several protocols have been developed and various biomarkers have been tested to predict the cause of SIRS and diagnose sepsis or bacteremia as early as possible. However, there is no ideal biomarker to date. Moreover, such markers as white blood cells (WBC), neutrophils count, CRP, or procalcitonin showed to be insufficiently sensitive to discriminate SIRS and sepsis, especially when used alone [19]. Lanziotti et al. stated that CRP alone is not specific especially during the first 24 h after the initial symptoms [20]. Additionally, early diagnosis of sepsis when fever is the only symptom is still complex.

Thus, our study was set out to investigate, in febrile children between 1 month to 5 years presented to ED, the diagnostic accuracy of used SIRS and sepsis markers, as WBC, neutrophils count, platelet count, CRP, NLR, PLR, MPV, as well as not so prevalent PLT/MPV. Also, we aimed to determine the best cut-off values, which could be implemented in clinical practice.

## 2. Methods and Materials

### 2.1. Study Design and Study Population

We conducted a retrospective, single-center pilot study in the Hospital of Lithuanian University of Health Sciences Kauno Klinikos. We analyzed case records of full-term (≥37 weeks at birth) previously healthy 1 month to 5 years of age febrile children who arrived at pediatric emergency department (PED) and were hospitalized to a pediatric department (PD) during 2011–2017. We included only patients with the final diagnosis of sepsis/bacteremia. Additionally, we selected a group of viral disease patients as a negative control group. Children with chronic diseases, immunodeficiency, or cancer; late arrival (>48 h from the onset of fever); or recent antibacterial therapy were not included in the analysis. Sepsis/bacteremia was determined by clinical symptoms (fever higher than 38 °C or lower than 36 °C, tachycardia or bradycardia, tachypnea), abnormal WBC, or more than 10% of immature neutrophil forms with or without a positive blood culture. Viral infection was diagnosed when clear viral infection such as nasopharyngitis, acute upper respiratory tract infection or pharyngitis were present. Further, all patients were divided into two groups—early and late arrival (less than 12 h and 12–48 h post the onset of fever, respectively). Moreover, we subdivided patients according to their age into infants (up to 12 months of age) and older children.

### 2.2. Laboratory Data

The following data were collected from the medical records—medical history including time from the onset of fever to the arrival to PED and physical examination results. Results of complete blood count (CBC), C-reactive protein (CRP), or blood culture were considered for the analysis. From the CBC, white blood cells (WBC), neutrophils, monocytes, platelets, and mean platelet volume (MPV) were collected. Additionally, neutrophil lymphocyte ratio (NLR) and platelet to mean platelet volume ratio (PLT/MPV) were calculated.

Permission to conduct the study was issued by Kaunas Regional Biomedical Research Ethics Committee (BE-2-54, on 7 September 2017) and the study was conducted in accordance with Declaration of Helsinki and good clinical practice guidelines.

### 2.3. Statistical Analysis

Data analysis was performed using Microsoft Excel and IBM SPSS Statistics version 21.0 software (SPSS Inc., Chicago, IL, USA) for Windows. The Shapiro-Wilk test was used to determine whether the data was normally distributed. Continuous variables are expressed as mean ± standard deviation or median and interquartile range, while qualitative data are presented as count and percentage. Continuous variables of two groups were compared by the paired samples *t*-test if the data were normally distributed, whereas Wilcoxon signed ranks and Mann—Whitney U tests were used to compare nonparametric data. Sensitivity and specificity were calculated for all the variables. A comparison of the diagnostic accuracy of inflammatory biomarkers was performed using receiver operating characteristics curves (ROC) analysis. A *p*-value of <0.05 was considered significant.

## 3. Results

**Figure 1:**
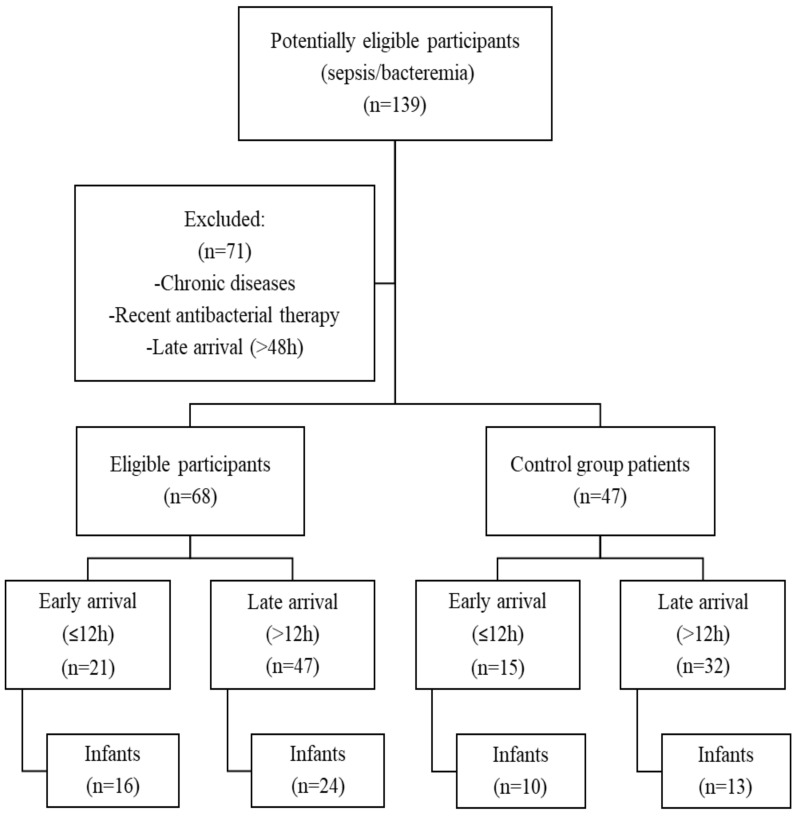
Study design and study population.

Between 2011 and 2017, we found 139 case records with at least two SIRS criteria and the final diagnosis of sepsis or bacteremia possibly eligible for our study. After excluding chronical diseases, recent antibiotic use, and late arrival (>48 h post onset of fever) cases, only 68 cases were submitted for further analysis. Then, we randomly selected 47 cases between 2011 and 2017 with clear viral infections (Figure 1). In all 115 children, the age median was 12.0 (4.0–24.0) months. There was no significant difference between the groups with respect to subject age and gender (9.0 (3.0–24.0) months versus 12.0 (6.0–27.0) months, *p* = 0.274 for age; and 31 (45.6%) versus 23 (48.9%), *p* = 0.857 for male gender).

Not surprisingly, all sepsis/bacteremia patients demonstrated significantly higher WBC (17.94 ± 10.04 × 10^9^/L versus 10.42 ± 4.21 × 10^9^/L; *p* < 0.001) and neutrophils count (10.93 ± 8.03 × 10^9^/L versus 5.08 ± 3.42 × 10^9^/L; *p* < 0.001), as well as CRP level (65.86 (20.80–133.69) mg/L versus 8.49 (2.11–20.85) mg/L; *p* < 0.001). Moreover, sepsis/bacteremia patients had a relevant increase in absolute platelets count (370.15 ± 134.65 × 10^9^/L versus 288.91 ± 107.14 × 10^9^/L; *p* = 0.001), NLR (2.69 ± 2.03 versus 1.83 ± 1.70; *p* = 0.006) and PLT/MPV (41.42 ± 15.86 versus 33.45 ± 17.97; *p* = 0.001) when compared with the viral group. Baseline characteristics of our study subjects are summarized in Table 1.

### 3.1. Early versus Late Arrival

Regarding the utility of these biomarkers to predict early sepsis/bacteremia, all participants were further divided into two groups—early (≤12 h from the onset of clinical symptoms) and late arrival (12–48 h) groups. The WBC count (16.11 ± 9.11 × 10^9^/L versus 9.89 ± 4.65 × 10^9^/L; *p* = 0.007), platelets count (390.43 ± 88.11 × 10^9^/L versus 282.29 ± 79.35×10^9^/L; *p* = 0.001) and CRP level (20.00 (7.95–65.22) mg/L versus 2.73 (1.00–13.89) mg/L; *p* = 0.008) were significantly elevated in sepsis/bacteremia children who arrived early when compared with the viral infection group. The NLR and MPV were similar in both groups, whereas PLT/MPV ratio was significantly higher in early arrival sepsis/bacteremia group (41.42 ± 15.86 versus 33.45 ± 17.97; *p* = 0.001).

Again, sepsis/bacteremia patients who arrived late into ED demonstrated significant increase in WBC count (18.70 ± 10.53 × 10^9^/L versus 10.76 ± 4.08 × 10^9^/L; *p* < 0.001) and CRP level (99.58 (43.92–182.30) mg/L versus 11.33 (6.11–23.40) mg/L; *p* < 0.001) when compared with viral group patients. However, the difference in platelets count and PLT/MPV ratio was not significant between the groups (*p* = 0.053 for platelets and *p* = 0.106 for PLT/MPV). In contrast to early arrival patients, late arrival sepsis/bacteremia group demonstrated significantly elevated neutrophils count (11.58 ± 7.85 × 10^9^/L versus 5.17 ± 3.20 × 10^9^/L; *p* < 0.001) and NLR (2.77 ± 2.00 versus 1.74 ± 1.60). The comparison of laboratory markers between early and late arrival bacterial and viral infections study participants are summarized in Table 2 and Table 3.

### 3.2. Infants Population

Of 115 case records who met our inclusion criteria, 63 (54.8%) were less than 12 months old. These infants were similarly further divided into early (≤12 h) and late (>12 h) arrival groups. Data analysis demonstrated significant difference in WBC count (12.80 ± 5.45 × 10^9^/L versus 8.59 ± 3.62 × 10^9^/L; *p* = 0.037) and platelets count (403.19 ± 84.03 × 10^9^/L versus 304.50 ± 80.29 × 10^9^/L; *p* = 0.010) between sepsis/bacteremia and viral group participants who arrived to ED during first 12 h after the onset of fever. There was no significant difference between the groups in neutrophils count, CRP level or NLR (*p* = 0.121 for neutrophils count; *p* = 0.120 for CRP level and *p* = 0.598 for NLR), while PLT/MPV ratio was significantly higher in sepsis/bacteremia group (42.70 ± 8.57 versus 31.01 ± 8.21; *p* = 0.008).

Similar to the entire study population, neutrophils count, CRP level and NLR were significantly elevated in sepsis/bacteremia patients who arrived late (>12 h after the onset of symptoms) (10.77 ± 7.67 × 10^9^/L versus 4.45 ± 2.70 × 10^9^/L, *p* = 0.003 for neutrophils count; 63.71 (28.45–128.25) mg/L versus 7.50 (4.57–14.05) mg/L, *p* < 0.001 for CRP level; and 1.90 ± 1.25 versus 1.16 ± 1.06, *p* = 0.025 for NLR). There was not significant difference in PLT/MPV between the groups (*p* = 0.316). The MPV was significantly lower in infants with viral infection and late arrival to ED when compared with sepsis/bacteremia group (8.94 ± 0.95 fL versus 9.44 ± 0.85 fL; *p* = 0.046). Comparison of infants’ laboratory markers between bacterial and viral infection patients are demonstrated in Table 4 and Table 5.

### 3.3. Prediction of Sepsis/Bacteremia

Among the laboratory biomarkers, CRP level, WBC count, PLT/MPV, and NLR were the best discriminators between sepsis/bacteremia patients and those with viral infection (Table 6 and Table 7). A value of 30.0 for PLT/MPV identified subjects with sepsis/bacteremia with the sensitivity of 76.1% and specificity of 46.8% was observed in the entire study population. The calculated NLR threshold of 1.58 demonstrated the sensitivity and specificity of 73% and 58%, respectively, for identifying children with sepsis/bacteremia. Subgroup analysis revealed that PLT/MPV was a more accurate biomarker in the infant population who arrived at PED within 12 h after the onset of fever when compared with NLR (sensitivity of 93.8%, specificity of 55.6% for PLT/MPV value of 32.0 versus sensitivity of 50.0%, specificity of 77.8% for NLR value of 1.35) (Table 8 and Table 9). In contrast, NLR value of 1.2 demonstrated a higher level of sensitivity and specificity in the late arrival infant subgroup when compared with PLT/MPV threshold of 40.0 (Table 8 and Table 9).

## 4. Discussion

Pediatric sepsis is a life threatening condition and one of the major health issues in infants and children [1,2,3,5]. This condition arises from the host response to infection. Patient outcomes are determined not only by virulence of invading pathogens, but also by host response, which can be highly damaging to own organs and tissues as host targets are not discriminated from the microbial ones [21]. Child’s immune system differs remarkably from an adult in terms of innate and adaptive immunity. Among all children, neonates and infants are the most immune-compromised. Their innate and adaptive immunity system responses are poorly developed [22,23,24]. The cumulative deficits in innate and adaptive immune system increase infants’ and young children’s susceptibility to develop severe infections from various pathogens [25,26]. Even though many pathogens have been eradicated due to vaccinations, nevertheless some of the most common sepsis inducing microorganisms—*Neisseria meningitidis*, causing meningococcemia, *Staphylococcus aureus* and *Streptococcus pyogenes*—remain, according to Randolph et al. [27]. Thus, early recognition of a pathogenic agent and early detection of sepsis is of high importance in infants and children. Hundreds of molecules have been proposed as biomarkers in sepsis [20,28]. However, only around 20% are assessed for sepsis diagnostics. And even less are reasonable and applicable in clinical settings. Moreover, in ED biological diagnostic markers must correspond to the local patient profile—must be easily accessible, rapid, sensitive, and specific as much as possible, and cheap. To date, most sepsis biomarkers are discussed in terms of sepsis prognosis and duration of antibiotic use [20,28,29]. Less has been investigated about early diagnosis of bacteremia. Most commonly used CRP, WBC, and neutrophil count, and lactate or procalcitonin (PCT) are not always sensitive and specific. Moreover, in terms of SIRS in early diagnostic settings, they fail to predict ongoing bacterial infection and/or sepsis when fever is the only symptom at presentation [30]. Some of the studies advise to use a combination of biomarkers as the sensitivity and specificity might increase than using a biomarker alone [31] Nevertheless, WBC and CRP remain the most widely used biomarkers in bacterial infection diagnostics. CRP is an acute phase protein released by the liver after the onset of inflammation or tissue damage. It peaks only at 24–48 h post infection onset [32,33,34,35]. Further, CRP can increase during minor infections. Therefore, it fails in early sepsis diagnostics, especially, among infants. No surprise, we observed a more significant increase in CRP levels among late arrived septic children (*p* < 0.001), rather than early (*p* = 0.008). As expected, CRP has shown no significant diagnostic value among early arrived infants (≤12 months). WBC is a part of innate immune response. These cells typically are one of the primary cells to respond to systemic infection [36], as well as, neutrophils, which are a part of polymorphonuclear leukocytes. Neutrophils marginate at the site of infection and take part in the bacterial removal [37]. According to our study, WBC counts were significantly higher at all time-points of arrival and in all study groups. However, significant raise in neutrophil counts was observed in bacterial group only at the late time of arrival (*p* < 0.001), when compared with early arrived children (*p* = 0.077).

Many clinical studies provide NLR (neutrophil to lymphocyte ratio) in addition to CRP, WBC, and neutrophil count, as an important marker in early bacteremia diagnostics and evaluation of sepsis outcomes [38,39,40,41,42]. Also, NLR was observed in some studies to be more efficient than regular inflammation biomarkers among adults [41,43]. Neutrophils are the key cell type of innate immunity and first line cellular defense cells against infectious agents. Lymphocytes play an important role in adaptive immunity. Immune response to different triggers reacts with an increase in neutrophil counts and lymphocyte count reduction. Thus, NLR was shown to be significant in adult population in early bacteremia prediction compared to standard diagnostic biomarkers [41,42,43]. Besides, according to few studies in infants and neonates, together with CRP, NLR is an accurate sepsis predictor [44,45]. We evaluated NLR as a potential biomarker for early sepsis diagnostics. However, it appeared to be significantly elevated only in late arrived children, as well as infants (≤12 months). Yet, precise standardized cut-off levels which could be used in clinical settings are still under debate leading to a complicated practical application of this biomarker. Gurol et al. evaluated the most sensitive and specific cut-off value of >5 in the adult population. However, NLR was proposed as a marker in terms of duration of antibiotic therapy [46]. Another study has suggested NLR threshold of 1.77 for the diagnosis of neonatal sepsis [44]. According to our study, the NLR cut-off value in early arrived infants was 1.35 with a sensitivity and specificity of 50% and 77.8%, respectively. However, in our study, 1.2 was the most sensitive and specific NLR value in infants who arrived late (see Table 8).

For a long time, platelets have been recognized for their roles in hemostasis and thrombosis. Lately, they have received an increased attention for their function in infectious diseases, inflammation and innate immunity [47,48,49,50]. Nowadays, platelets are identified as first-line indicators in detecting and acting to different pathogens. They are one of the first to respond to the damage signals in the vasculature and in the extracellular space [48,51,52,53]. Data have shown that during the early phases of bacterial infection, there is an enormous increase in platelet count in the bloodstream, which later decreases excessively [44]. Agrawall et al. observed thrombocytopenia in pediatric patients, associating it with poor outcomes [54]. However, the mechanism of low platelets is not fully understood, although it is believed that endothelial damage during sepsis induces bone marrow to remove all of the platelets reserve [55]. During viral infection, platelets interact with leukocytes and mediate immune response [56]. Our study presented that PLT counts are significantly elevated in early (<12 h) arrived children and infants (younger than 12 months). But they have no significance in pediatric patients, who arrived at ED > 12 h after the onset of first symptoms of the disease. With a cut-off value of 300 × 10^9^/L, platelet count was 71.7% sensitive and 54.5% specific to discriminate between two study groups with moderate AUC of 0.677 (*p* = 0.001). Platelet function correlates with their size, as larger platelets are more reactive than normal. Morrel et al. has observed that platelets are highly active in early phase of inflammation through their granules. These granules store large variety of proteins and mediators, interleukins such as interleukin-1β (IL-1β), different chemokines, or pre-messenger RNA [49,57,58,59,60]. Following platelet stimulation, granules undergo exocytosis and release their content into extracellular environment. As a result, activated platelets affect locally, for instance triggering an endothelial reaction, or systemically, inducing CRP production. The specific indices which reflect the size of thrombocyte are mean platelet volume (MPV). Increased MPV signifies the release of larger and younger platelets into the circulation. Few studies indicated changes in MPV could be used as a considerable prognostic factor of mortality in patients with sepsis [61,62]. No significant changes were found in MPV in early arrived children of both study groups. While late arrived infants (<12 months) with acute viral infection had relevantly decreased MPV when compared with septic children (*p* = 0.046). We found no significant differences in other platelet markers as PDW or PLR (data not shown). Considering early activation of platelets and following changes in their volume, we analyzed platelet to MPV ratio. To date, there are only few studies done to prove PLT markers’ significance among other diseases, but sepsis [63,64]. Most of the studies analyze MPV to PLT ratio as a marker of the disease outcome. Few studies among adults have suggested that MPV/PLT could be used as an early prognostic factor for severe sepsis [65,66,67]. However, no studies were done in a pediatric population. Moreover, there is no data on early septic diagnostics in infants ≤12months of age. To our knowledge, this is the first study to investigate and show PLT/MPV value in early pediatric sepsis/bacteremia diagnosis, especially in infants. With an AUC of 0.676 (CI 0.575–0.776, *p* = 0.002), PLT/MPV has shown to be a sensitive marker (76.1%) at a cutoff value of 30.0 to differentiate between viral infection and sepsis/bacteremia in early arrived children. Moreover, the value of PLT/MPV was higher in septic infants who arrived up to 12 h after the onset of fever compared to infants with viral respiratory illness (42.70 ± 8.57 versus 31.01 ± 8.21 respectively, *p* = 0.008). This marker with a cut-off value of 32.0 showed to be highly sensitive (93.8%) to distinguish between early arrived sepsis/bacteremia group versus infants with viral disease.

### Limitations

There are a few limitations of this study which must be considered. First limitation we see that it is a retrospective analysis of already documented data. To prove if these data have prognostic value in early prediction of severe bacterial infection, a perspective study should be performed. Secondly, our study is limited only to a group of septic children and clear viral infection. Thus, all SBI children could be included and previously described biomarkers should be compared to a viral diseases’ population. Thirdly, as there is no data regarding cut-off values of those biomarkers in children, control group of healthy children could be of a benefit for the reference values. Further, we did not include children with comorbidities which can lead to impaired immune system (as diabetes, neurological disorders, chronic respiratory diseases, and others). We do think, another study could be done including these conditions.

## 5. Conclusions

Our data revealed that the most commonly used WBC, CRB, and neutrophil count as well as PLT were sensitive to predict sepsis/bacteremia overall. Additionally, NLR and PLT/MPV could help discriminate between SIRS caused by viral disease or sepsis. NLR and PLT/MPV performed better as an early sepsis prediction marker in all children compared to WBC, CRP, or PLT count. Most importantly, it was a more sensitive indicator to distinguish between viral and bacterial cause of SIRS in children ≤12 months of age than standard infection prediction markers like WBC, CRP, or neutrophil count. No exceptional data was observed among late arrival group (>24 h) as WBC, CRP, and neutrophil count showed to be most sensitive biomarkers in sepsis/bacteremia group. Nevertheless, NLR could aid in differential diagnosis of SIRS and together with MPV could be beneficial especially in infants (≤12 months).

## Figures and Tables

**Table 1:** Baseline characteristics of the entire population.

Parameter	Bacterial (*n* = 68)	Viral (*n* = 47)	*p*-Value
Demographic Data			
Age, months	9 [3–24]	12 [6–27]	0.274
Male gender, *n* (%)	31 (45.6)	23 (48.9)	0.857
Laboratory Markers			
WBC, ×10^9^/L	17.94 ± 10.04	10.42 ± 4.21	<0.001
Neutrophils, ×10^9^/L	10.93 ± 8.03	5.08 ± 3.42	<0.001
PLT, ×10^9^/L	370.15 ± 134.65	288.91 ± 107.14	0.001
CRP, mg/L	64.86 [20.80–133.69]	8.49 [2.11–20.85]	<0.001
NLR	2.69 ± 2.03	1.83 ± 1.70	0.006
MPV	9.03 ± 1.21	9.16 ± 1.33	0.717
PLT/MPV	41.42 ± 15.86	33.45 ± 17.97	0.001

Results are expressed as mean ± standard deviation, median (range) or *n* (percentage). WBC = white blood cells; PLT = platelets; CRP = C-reactive protein; NLR = neutrophil-lymphocyte ratio; MPV = mean platelet volume; PLT/MPV = platelet and mean platelet volume ratio.

**Table 2:** Comparison of laboratory markers between bacterial and viral infections patients (early arrival).

Laboratory Marker	Bacterial (*n* = 21)	Viral (*n* = 15)	*p*-Value
WBC, ×10^9^/L	16.11 ± 9.11	9.89 ± 4.65	0.007
Neutrophils, ×10^9^/L	9.43 ± 8.59	5.06 ± 4.07	0.077
PLT, ×10^9^/L	390.43 ± 88.11	282.29 ± 79.35	0.001
CRP, mg/L	20.00 (7.95–65.22)	2.73 (1.00–13.89)	0.008
NLR	2.58 ± 2.19	2.07 ± 2.00	0.547
MPV	9.02 ± 1.09	9.58 ± 0.82	0.202
PLT/MPV	43.86 ± 11.58	29.80 ± 8.69	<0.001

Abbreviations as in Table 1.

**Table 3:** Comparison of laboratory markers between bacterial and viral infections patients (late arrival).

Laboratory Marker	Bacterial (*n* = 47)	Viral (*n* = 36)	*p*-Value
WBC, ×10^9^/L	18.70 ± 10.53	10.76 ± 4.08	<0.001
Neutrophils, ×10^9^/L	11.58 ± 7.85	5.17 ± 3.20	<0.001
PLT, ×10^9^/L	354.17 ± 144.81	291.41 ± 119.84	0.053
CRP, mg/L	99.58 (43.92–182.30)	11.33 (6.11–23.40)	<0.001
NLR	2.77 ± 2.00	1.74 ± 1.60	0.003
MPV	9.03 ± 1.28	8.93 ± 1.46	0.640
PLT/MPV	40.31 ± 17.47	35.17 ± 20.87	0.106

Abbreviations as in Table 1.

**Table 4:** Comparison of infants’ (≤12 months) laboratory markers between bacterial and viral infection patients (early arrival).

Laboratory Marker	Bacterial (*n* = 16)	Viral (*n* = 10)	*p*-Value
WBC, ×10^9^/L	12.80 ± 5.45	8.59 ± 3.62	0.037
Neutrophils, ×10^9^/L	5.83 ± 4.09	3.49 ± 2.28	0.121
PLT, ×10^9^/L	403.19 ± 84.03	304.50 ± 80.29	0.010
CRP, mg/L	15.86 (2.51–28.03)	2.02 (1.22–15.89)	0.120
NLR	1.64 ± 1.43	1.33 ± 1.20	0.598
MPV	9.44 ± 0.62	9.78 ± 0.44	0.152
PLT/MPV	42.70 ± 8.57	31.01 ± 8.21	0.008

Abbreviations as in Table 1.

**Table 5:** Comparison of infants’ (≤12 months) laboratory markers between bacterial and viral infections patients (late arrival).

Laboratory Marker	Bacterial (*n* = 24)	Viral (*n* = 13)	*p*-Value
WBC, ×10^9^/L	19.74 ± 11.65	11.11 ± 4.41	0.015
Neutrophils, ×10^9^/L	10.77 ± 7.67	4.45 ± 2.70	0.003
PLT, ×10^9^/L	419.30 ± 155.35	338.49 ± 150.28	0.106
CRP, mg/L	63.71 (28.45–128.25)	7.50 (4.57–14.05)	<0.001
NLR	1.90 ± 1.25	1.16 ± 1.06	0.025
MPV	9.44 ± 0.85	8.94 ± 0.95	0.046
PLT/MPV	44.80 ± 17.28	39.45 ± 22.10	0.316

Abbreviations as in Table 1.

**Table 6:** Sensitivity and specificity of the laboratory markers (entire population).

Laboratory Marker (Cut-Off Value)	Sensitivity, %	Specificity, %
WBC (>9.5 × 10^9^/L)	77.6	58.7
Neutrophils (>5.0 × 10^9^/L)	71.6	43.5
PLT (>300 × 10^9^/L)	71.7	54.5
CRP (>20 mg/L)	80.3	68.8
NLR (1.58)	73.0	57.7
MPV (9.0)	68.7	34.0
PLT/MPV (30.0)	76.1	46.8

Abbreviations as in Table 1.

**Table 7:** Area under the curve and confidence intervals for laboratory markers (entire population).

Laboratory Marker	AUC	95% CI	*p*-Value
WBC	0.739	0.647–0.830	<0.001
Neutrophils	0.736	0.644–0.828	<0.001
PLT	0.677	0.578–0.776	0.001
CRP	0.830	0.753–0.906	<0.001
NLR	0.650	0.545–0.754	0.007
MPV	0.489	0.381–0.597	0.842
PLT/MPV	0.676	0.575–0.776	0.002

Abbreviations as in Table 1.

**Table 8:** Sensitivity and specificity of laboratory markers for the infant population.

Laboratory Marker (Cut-Off Value)	Sensitivity, %	Specificity, %
Infants arrived at ED within 12 h		
NLR (1.35)	50.0	77.8
PLT/MPV (32.0)	93.8	55.6
Infants arrived at ED after 12 h		
NLR (1.2)	66.7	71.4
PLT/MPV (40.0)	56.5	64.3

Abbreviations as in Table 1.

**Table 9:** Area under the curve and confidence intervals for laboratory markers (infant population).

Laboratory Marker	AUC	95% CI	*p*-Value
Infants arrived at ED within 12 h			
NLR	0.569	0.331–0.808	0.571
PLT/MPV	0.826	0.662–0.991	0.008
Infants arrived at ED after 12 h			
NLR	0.720	0.550–0.890	0.025
PLT/MPV	0.599	0.405–0.794	0.316

Abbreviations as in Table 1.
